# Artery of Percheron Infarction: A Short Review

**DOI:** 10.1177/2324709619867355

**Published:** 2019-08-09

**Authors:** Asim Kichloo, Shakeel M. Jamal, El-Amir Zain, Farah Wani, Navya Vipparala

**Affiliations:** 1 St. Mary’s Hospital, Saginaw, MI, USA; 2 Central Michigan University, Saginaw, MI, USA

**Keywords:** artery of Percheron, infarct, thalamic infarction

## Abstract

One uncommon type of ischemic stroke is occlusion of the artery of Percheron (AOP) leading to infarction of the paramedian thalami and mesencephalon. There are several variants of thalamic blood supply, and identifying the potential presence and infarction of an AOP is important in diagnosis and treatment of ischemic strokes affecting the thalami and mesencephalon, especially because of the unusual and variable presentation of these forms of ischemic strokes. This short review includes and discusses the case of a 58-year-old woman with an AOP infarct and indicates the importance of recognizing an AOP infarct early despite its clinical variations in order to treat the stroke in a timely fashion. This short review also includes a discussion of imaging modalities in such cases and clinical differential diagnoses to consider with management strategies.

## Introduction

**Figure 1.**
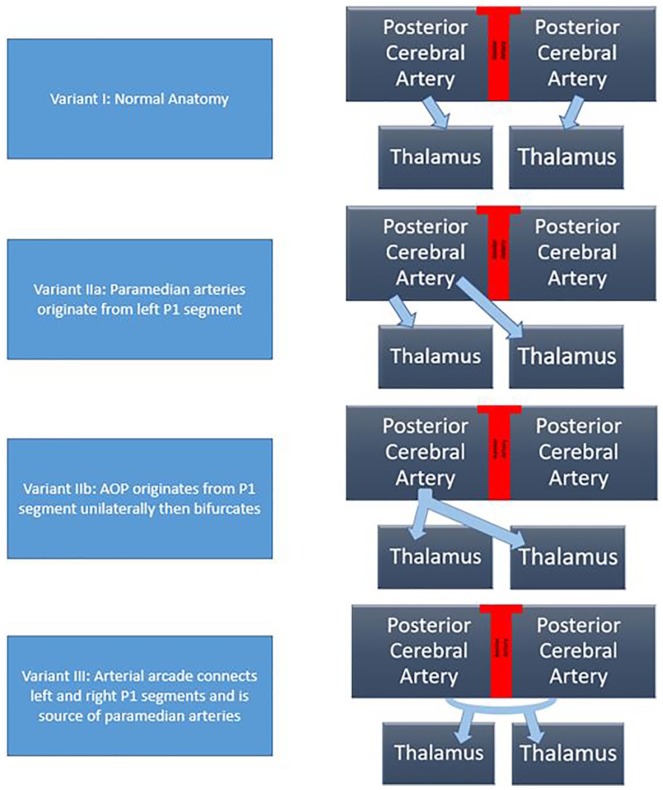
Variants of thalamic blood supply.

The thalami and the midbrain receive blood supply from perforating branches of posterior cerebral and communicating arteries.^1^ The thalami have blood supply categorized into 4 territories: anterior, paramedian, posterior, and inferolateral.^2-4^ The paramedian territories of the thalami are supplied by perforating thalamic arteries of posterior circulation known as paramedian arteries.^5^ There are 4 normal variants of neurovascular supply to the thalami and midbrain (Figure 1).^2^ The most common variant is variant I, which is where the perforating branches arise from the right and left posterior cerebral arteries (PCAs) individually. Variant IIa is where the left P1 segment is the source of both paramedian arteries. In variant IIb, the perforating arteries arise from the artery of Percheron (AOP). In these variants, the AOP comes from a part of the posterior cerebral artery, namely, the P1 segment. The AOP supplies the paramedian thalami and the rostral midbrain. Variant III is known as an arcade variant. In this anatomical variant, arcade gives off small perforating branches from one arterial arc. This arterial arc bridges the P1 segments and the PCAs together.^1-3^

Occlusion of the AOP may lead to an infarction of the paramedian thalami and mesencephalon.^6-9^ The AOP variant is present in 4% to 12% of the population.^4^ In 2 large stroke series studies, characteristic AOP infarct patterns constituted 0.1% to 2% of all ischemic strokes, indicating that this type of ischemic stroke is quite rare.^10^

## Case Presentation

A 58-year-old female with a past medical history of hypertension that was well controlled with medications and type 2 diabetes mellitus who recently lost her husband and father within a span of 2 weeks and was in a lot of stress was found by the police in a confused state at a general store, which resulted in the emergency medical services being called. On arrival of the emergency medical services, the patient became obtunded and was brought to the emergency room. Her last known contact with any family member was the night before in the evening with her niece over a phone conversation. She was maintaining her airways and saturating well throughout her course. Imaging with a head computed tomography (CT) and head and neck CT angiography (CTA) was done, both of which showed no evidence of acute stroke or any aneurysms. The patient was found to be in a hypertensive emergency with a blood pressure of 230/110 mm Hg. The patient was admitted to the neuro intensive care unit for further monitoring and treatment. Her metabolic panel was normal, and she had normal blood sugars. Straight catheterization, a urinalysis, and a urine drug screen were done. The urine drug screen came back negative.

**Figure 2.**
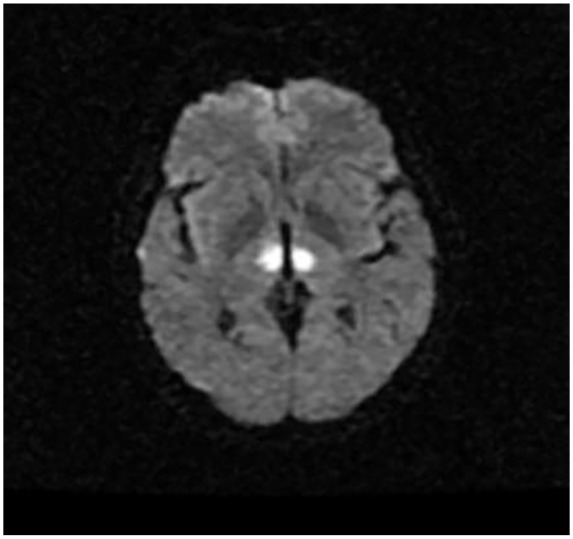
Diffusion-weighted axial MR showing diffusion restriction of the bilateral paramedian thalami.

**Figure 3.**
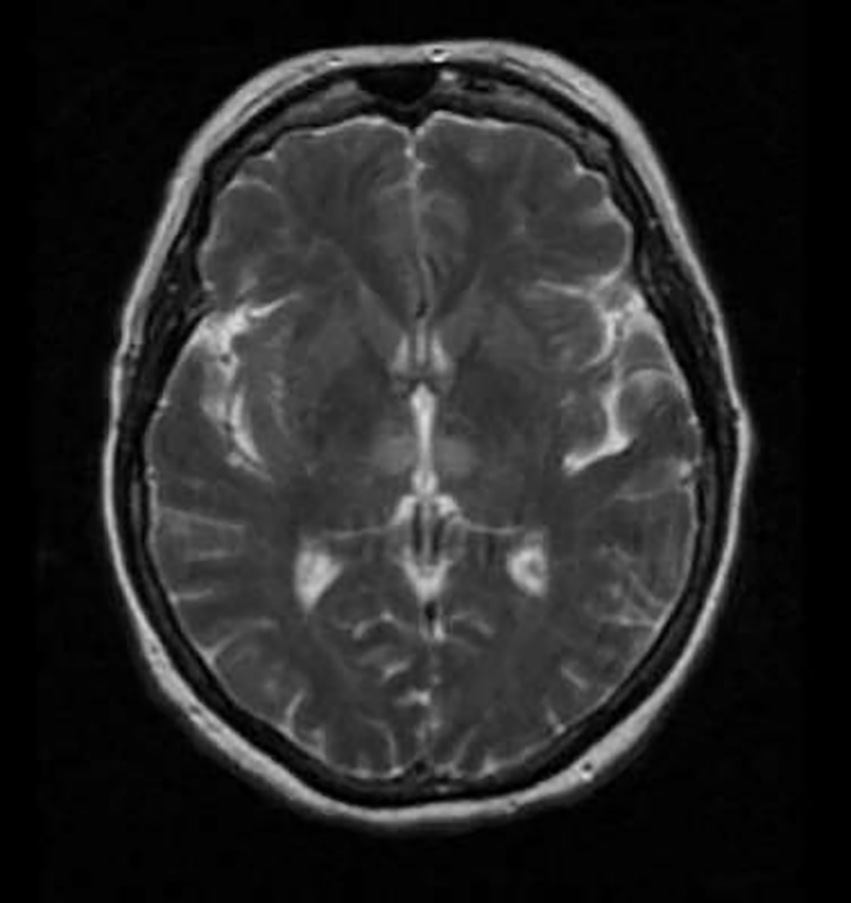
T2/FLAIR signal weighed axial MR demonstrating hyperdensities of bilateral paramedian thalami.

**Figure 4.**
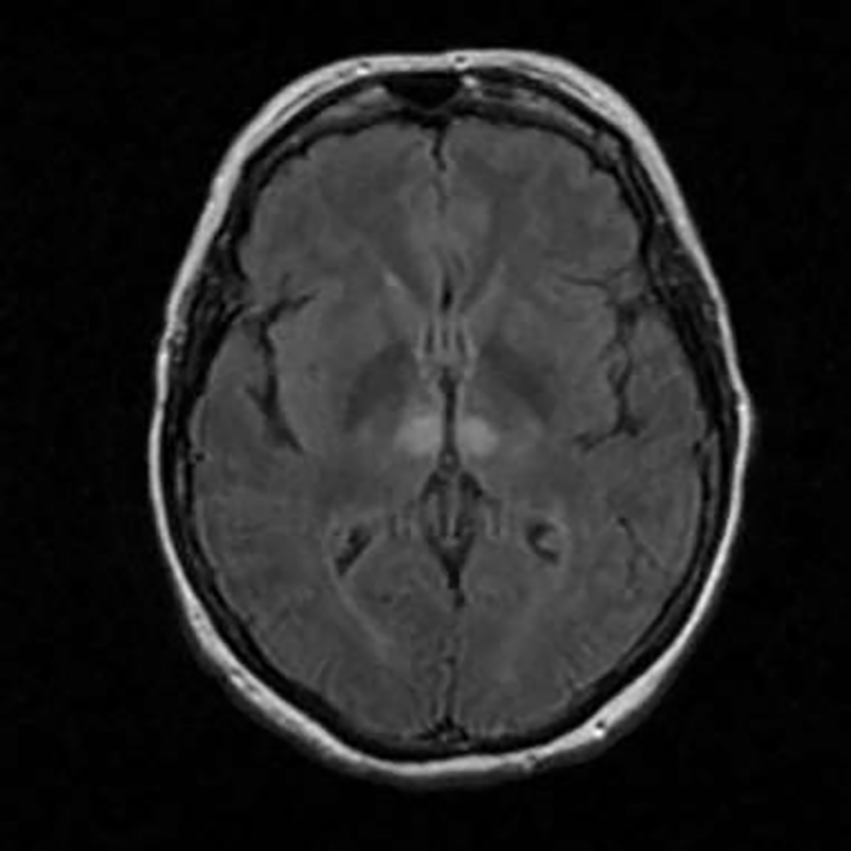
T1/FLAIR signal weighed axial MR demonstrating hyperdensities of bilateral paramedian thalami.

On examination, the patient was obtunded with a minimal response on deep sternal rub and on painful stimulus. Neurological examination revealed a positive gag reflex with a fixed and dilated left pupil at 7 mm with a normal nonreactive right pupil. The patient also had lateral deviation of the left eye with no nystagmus appreciated. Based on the lateral deviation of the left eye and clinical presentation, a magnetic resonance imaging (MRI) of the head was done. The MRI showed increased FLAIR (fluid-attenuated inversion recovery) signal within the periventricular white matter consistent with paramedian thalamic nuclei without midbrain involvement and acute bilateral ischemic infarction consistent with the AOP occlusion was diagnosed (Figures 2-4).

Because of her hypertensive emergency and more than 24 hours of documented stroke diagnosis since she was known at her baseline, the patient was not a candidate for tissue plasminogen activating factor (tPA). The neurosurgery recommendation was conservative management. The patient was kept in the neuro intensive care unit and got a 2-dimensional echocardiogram with bubble study done the next day, which was negative. Telemetry did not show any abnormal cardiac rhythms. The patient continued to improve. Within the first 48 hours, the patient was confused but awake and following minimal commands. She continued to show improvement and passed a speech and swallow test. Eventually, the patient was discharged to subacute rehab with neurological follow-ups and was given dual antiplatelet therapy with Aspirin and Plavix as per recommendations of neurosurgery with reevaluation for the need of dual antiplatelet in 4 weeks.

## Discussion

In one of the largest case series on the topic, 4 distinct variants of AOP infarcts were identified. One variant is infarction of the bilateral paramedian thalami and rostral midbrain. This variant made up about 43% of cases.^4^ Bilateral paramedian thalamic infarction without the involvement of midbrain is another variant, and this encompassed 38% of cases. Bilateral paramedian and anterior thalamic infarction with midbrain involvement made up 14% of cases. Finally, bilateral paramedian and anterior thalamic infarction without midbrain involvement was the least prevalent, encompassing only 5% of cases.^4^ In our patient, the MRI showed paramedian thalamic infarcts without the involvement of the midbrain, which is the rarest form of AOP infarction.

### Differential Diagnosis

There can be other types of infarcts that may result in bilateral thalamic lesions apart from the AOP infarcts; these include deep cerebral venous thrombosis as well as top of basilar syndrome. Top of basilar syndrome was ruled out in this patient as in top of the basilar artery syndrome, territories supplied by posterior cerebral, superior cerebellar, and pontine arteries are also involved when basilar artery is occluded in addition to bilateral paramedian thalami.^11,12^ Another presentation that may have thalamic infarction is venous sinus thrombosis, which is a result of occlusion of internal cerebral veins. Symptomatically, cerebral venous thrombosis may present with headache, vomiting, and papilledema and will result into infarction that can present with seizures, focal neurological deficits, and aphasia.^13^ Wernicke’s encephalopathy is also included in the differential diagnosis because it results in lesions in bilateral thalami, periaqueductal gray tectal plate, dorsal medulla, and mammillary bodies.^14^ Infections and osmotic myelinosis may be included in the differential diagnosis, as well.^13^

### Clinical Presentation Variability

Clinical features are quite variable in AOP infarcts and can be described in 7 patterns:

1. Mental status changes, including somnolence, stupor, and coma.
2. Behavior and memory impairment, which includes confusion, agitation, apathy, disinhibition, hyperphagia, amnesia, and pseudobulbar affect (a condition that is characterized by episodes of sudden uncontrollable and inappropriate laughing or crying).
3. Aphasia/dysarthria/slurred speech.
4. Ocular movement abnormalities that can include horizontal, vertical gaze paresis with or without pupillary involvement.
5. Motor deficits.
  - Note: this is defined as paresis or paralysis of any type affecting the face, upper limbs, or lower limbs.
6. Cerebellar signs and symptoms like ataxia and dysmetria.
7. Other nonspecific clinical presentations, such as hypersomnia, tremors, seizures, and hyperthermia.^12^

Clinical presentations may vary based on types of infarcts. First, bilateral paramedian thalamic with rostral bilateral paramedian and anterior thalamic infarct without midbrain involvement both result in motor symptoms disorder, oculomotor dysfunction, and aphasia. Seventy-five percent of these patients are reported to have unfavorable outcomes, and 25% have favorable outcomes. Second, bilateral paramedian thalamic infarct without midbrain involvement has clinical manifestations of behavioral and amnesic and motor impairment. Sixty-seven percent of these patients had favorable outcomes.^15^

Artery of Percheron infarcts should also be kept in the differential diagnoses when elderly patients present with an altered mental status. Patients with bilateral paramedian stroke with AOP may present with memory impairment, which was found in 58% of patients in one study; coma, which was found in 42% of patients; confusion, which was found in 53% of patients; or vertical gaze palsy, which was found in 65% of patients.^10^ Thalamic dementia, which develops from bilateral thalamic infarcts (not unilateral infarcts), results in memory impairment, confusion, and coma. This is because the thalamus has the reticular activating system, and paramedian thalamic infarct leads to disconnection of the thalamus and the cortex.^6^ As stated in one of the case series, paramedian thalamic infarcts present with behavioral, amnesic, and motor symptoms. This patient’s presentation was different, as she also presented with lateral deviation of the left eye and dilation of the left pupil. The presentation of isolated ophthalmoplegia is unique in our case, as ocular symptoms typically present with involvement of anterior thalamus and mesencephalon.

### Workup

In this case, hypertensive encephalopathy, an obtunded initial presentation of the patient and lateral deviation of the left eye with dilation of left pupil promoted MRI scan, which led to the identification of paramedian infarcts. Thus, it is very important to do a detailed neurologic examination in a patient’s workup. Multiple imaging modalities might be required to properly diagnose posterior circulation infarcts, including CT, CT perfusion, CTA, MRI, diffusion-weighted imaging, and FLAIR. Close follow-up clinical examinations are very critical, as well.^10^

The imaging workup of choice for diagnosing AOP infarct early are FLAIR and diffusion-weighted imaging.^4,12^ An initial normal MRI brain cannot rule out AOP ischemic infarcts.^2^ Therefore, a repeat MRI focused on vertebrobasilar territory should be considered.^3^ CT perfusion can be helpful, as it can help delineate the areas of the brain that can be salvaged by interventions from the areas that are destined to get infarcted regardless of intervention.^12^ In other words, identification of infarcted areas is easier with perfusion CTs as compared with non-contrast CT in cases of AOP infarcts.

Conventional angiography is not used to diagnose occluded AOP, which is because the inability to image AOP using conventional angiography does not rule out its presence. This is because it may not be visualized if it is occluded.^3,12^ In our patient, a head CT and CTA of the head and neck were done and showed no evidence of acute stroke, any thromboembolic events, or any aneurysms. Our patient had lateral deviation of the left eye, leading the medical team to do an MRI scan of the head. The MRI showed increased FLAIR signal within the periventricular white matter, which is consistent with bilateral paramedian thalamic infarcts.

### Management

Management of AOP ischemic stroke remains a difficult task due to several factors. There is high P1 PCA anatomic variability, and as mentioned above, type IIb AOP is found in 4% to 18% of the population; however, this could be an underestimation.^10^ The anatomic variability and incidence paired with the variability of presenting symptoms may mean that physicians are unaware of this type of stroke and its associated clinical features. AOP occlusions also present a neurodiagnostic challenge because the small vessel size makes the finding rare on digital angiography. Some report records only 4 cases of one identified AOP variant.^4^ Therefore, a proper diagnosis of an AOP occlusion is currently a major obstacle.

Generally, the standard of care for the treatment of an acute ischemic stroke depends on timing, location of lesion, and contraindications to use of thrombolytics. Recombinant tPA administered within 4.5 hours of onset combined with mechanical thrombectomy within 6 hours is argued to be the preferred treatment of proximal cerebral artery occlusion.^16^ The American Heart Association has similar guidelines regarding stroke occlusion of the proximal middle cerebral or internal carotid artery. When tPA is contraindicated, patients may still benefit from mechanical removal.^17^ Since AOP diagnosis is often delayed, implementation of this treatment recommendation is difficult.

Along similar lines, AOP represents a small diameter, distal artery of the posterior circulation, and information on the effectiveness of mechanical removal is difficult to locate. Endovascular revascularization is seldom used an option in AOP infarcts, as it is too small to be visualized.^17^

One review includes 2 different treatment plans for AOP occlusions. Emergent cases of AOP stroke should initially be treated with intravenous (IV) heparin and tPA if not contraindicated followed by subsequent long-term anticoagulation. Nonemergent cases not involving the midbrain may be treated by rehabilitation and unspecified oral anticoagulation, which is administered mainly to prevent future obstructions. Those involving the midbrain are treated with IV heparin.^18^ Reports of successful recovery have been reported with IV heparin use, but further investigation is likely necessary to create a standard but optimal treatment plan.^19^ Another case reports the administration of IV heparin with the activated clotting time maintained between 300 and 350 seconds with a follow-up MRI of the brain, which has shown minimal residual stroke with administration of IV heparin.^20^ Last, treatment of AOP infarction should be directed toward the pathophysiology of underlying disease process. Long-term oral anticoagulant and antiplatelet therapy with Aspirin and Plavix is a matter of debate and are usually directed by clinicians depending on the underlying disease process like cardioembolic events require long-term oral anticoagulants whereas cryptogenic causes require antiplatelet therapy.

### Management in Our Patient

Since the patient in this case was hypertensive with a systolic blood pressure above 230 mm Hg, tPA could not be given. Nicardipine was started to control the blood pressure in a tight range and allowing permissive hypertension for first 48 hours. The patient’s condition improved over the next 48 hours and was confused but awake. AOP infarcts result from occlusion of small vessels and cardiac embolization.^21,22^ However, no identifiable cause of AOP infarct could be identified in our patient. Thus, the stroke was reported to be a cryptogenic stroke. A transthoracic echocardiography was negative for intracardiac embolism and patent foramen ovale. Cardiac telemetry was negative over the hospital stay. On the third day, the patient passed the speech and swallow test and continued to show improvement. The patient was eventually discharged to subacute rehab with neurological follow-ups and dual antiplatelet therapy of Aspirin and Plavix.

## Conclusion

Artery of Percheron obstruction is a rare form of ischemic stroke, but recognition of the possible presence of an AOP obstruction both clinically and in imaging is essential to the administration of time-sensitive treatments, such as mechanical removal of the obstruction or tPAs. Prompt diagnosis allows for earlier treatment, which positively affects patient outcomes. AOP infarcts may present with a wide array of symptoms and are hard to identify using standard imaging modalities, making diagnosis quite challenging. Keeping AOP infarcts on the differential diagnosis in patients with a variety of symptoms may be beneficial to patients.
